# A sort and sequence approach to dissect heterogeneity of response to a self-amplifying RNA vector in a novel human muscle cell line

**DOI:** 10.1016/j.omtn.2024.102400

**Published:** 2024-11-26

**Authors:** Rachel D. Barton, John S. Tregoning, Ziyin Wang, Daniel Gonçalves-Carneiro, Radhika Patel, Paul F. McKay, Robin J. Shattock

**Affiliations:** 1 Department of Infectious Disease, Imperial College London, London W2 1PG, UK; 2 National Heart and Lung Institute, Imperial College London, London W2 1PG, UK

**Keywords:** MT: Delivery Strategies, RNA, vaccine, expression, innate immunity, self-amplifying, alphavirus

## Abstract

Self-amplifying RNA (saRNA) is an extremely promising platform because it can produce more protein for less RNA. We used a sort and sequence approach to identify host cell factors associated with transgene expression from saRNA; the hypothesis was that cells with different expression levels would have different transcriptomes. We tested this in CDK4/hTERT immortalized human muscle cells transfected with Venezuelan equine encephalitis virus (VEEV)-derived saRNA encoding GFP. Cells with the highest expression levels had very high levels of transgene mRNA (5%–10% total reads); the cells sorted with low and negative levels of GFP protein also had detectable levels of both VEEV and GFP RNA. To understand host responses, we performed RNA sequencing. Differentially expressed gene (DEG) patterns varied with GFP expression; GFP high cells had many more DEGs, which were associated with protein synthesis and cell metabolism. Comparing profiles by an unsupervised approach revealed that negative cells expressed higher levels of cell-intrinsic immunity genes such as *IFIT1*, *MX1*, *TLR3*, and *MyD88*. To explore the role of interferon, cells were treated with the Jak inhibitor ruxolitinib. This reduced the number of DEGs, but differences between cells sorted by expression level remained. These studies demonstrate the complex interplay of factors, some immune related, affecting saRNA transgenes.

## Graphical abstract

**Figure:**
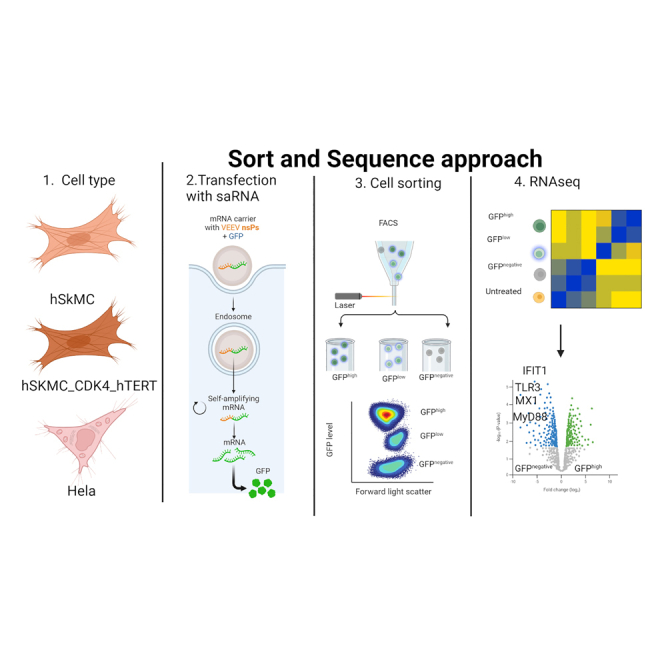

## Teaser

Shattock and colleagues used a sort and sequence approach to identify factors that influence the expression of transgenes from self-amplifying RNA as a way to understand the vaccine platform. We observed a very high level of transgene transcripts (10%) as a proportion of total RNA in positive cells and altered transcriptomes.

## Introduction

RNA is a potent platform for gene delivery, both vaccine antigens and therapeutic proteins, although there are necessarily differences in the interaction of the immune system with the expressed proteins. Self-amplifying RNA (saRNA) offers a considerable advantage over non-replicating mRNA in terms of expressed protein yield. However, further optimization is required to maximize expression from this promising platform. Understanding factors in transfected cells that influence saRNA replication and translation is important to maximize protein yield. One consideration is how the cell senses and reacts to foreign RNA.^1^ In particular, interferon (IFN) signaling, which induces an antiviral state in cells, triggers the inhibition of translation and degradation of viral RNA.^2^ Virally derived saRNA constructs may be especially sensitive to type I IFN and downstream IFN-stimulated genes (ISGs), and therefore, targeting these might increase expression.

Different cell types have different patterns of intrinsic response, which may affect the expression of RNA transgenes; thus, it is important to examine the cells that are targeted by vaccination. The most common route of delivery for RNA is into the muscle, particularly in the context of vaccination. Understanding how muscle cells contribute to immunity, particularly which cells in the muscle tissue are responsible for the expression of antigens from transfected nucleic acid and whether muscle cells play any other active roles in the immune response will be instructive. Both myocytes and infiltrating immune cells are capable of expressing large quantities of antigen.^3^^,^^4^^,^^5^ It is believed that saRNA expression in myocytes leads to apoptosis; the resultant apoptotic bodies are then phagocytosed by antigen-presenting cells (APCs) for antigen presentation in the lymph nodes.^6^

To a large degree, RNA candidate design and down-selection is performed *in vitro*. It is vital, therefore, to use the most appropriate cell types during early development, ensuring that decisions about vaccine candidate performance are made in a relevant cellular context. Given that muscle cells play a key role in the synthesis of antigens from saRNA at the site of injection,^5^^,^^7^ it is important that we utilize muscle cells during vaccine development, particularly in early-stage *in vitro* studies. However, their complicated biology can make studying muscle cells more difficult *in vitro* as the highly organized structure of muscle fibers cannot be easily replicated in cell culture.^8^ Alternatively, a simpler form of the multinucleated muscle fibers, called myotubes, can be stimulated to form from myoblasts in tissue culture; however, this step represents terminal differentiation, and the cells will no longer divide once differentiated.^8^ Conveniently, the literature suggests that myoblasts are a major source of antigen expression from nucleic acid vaccines,^9^ meaning that myoblasts represent a good screening platform.

No healthy human myoblast cell line is commercially available. Instead, it is common practice to isolate myoblasts directly from muscle biopsies by dissociating the tissue with enzymes.^10^ Although these primary myoblasts are capable of expansion *in vitro*, there are several limitations to their use, including limited proliferative capacity (typically only 15 population doublings), phenotypic drift, and contamination with other muscle-derived cells, most often fibroblasts.^11^ It is possible to overcome some of these limitations through the immortalization of the myoblasts with the human telomerase catalytic subunit, hTERT, which maintains telomere length, and CDK4, which blocks the p16^INK4a^-dependent stress pathway.^11^^,^^12^ Using lentiviral vectors to insert *CDK4* and *hTERT* genes into the genome of primary human myoblasts has been shown to generate immortalized cell populations.^13^^,^^14^

We derived a novel CDK4-hTERT myoblast cell line and then transfected cells with a Venezuelan equine encephalitis virus (VEEV)-derived saRNA expressing GFP. As a comparison we used HeLa cells alongside the muscle cells. HeLa cells are a popular human IFN-competent cell line, originally isolated from a sample of cervical cancer.^15^ At a cellular level, there is a heterogeneity of responses following RNA transfection, which provides a window to dissect why some cells express transgenes and others do not. We used a sort and sequence approach to compare differences in host transcription between cells that express transgenes and those that do not. Cells from the same well were sorted based on their GFP expression level and bulk RNA sequencing (RNA-seq) performed. Cells not expressing GFP had detectable but significantly lower levels of GFP mRNA, but there was no difference between low- and high-expressing cells, suggesting a role for the host response. When the transcriptomes between high and low expressor cells were compared, we observed differences in gene upregulation following transfection. We also explored the impact of the JAK/Stat inhibitor ruxolitinib and the transcriptional profile and compared patterns in human and murine cells.

## Results

### Generation of immortalized muscle cells

**Figure 1:**
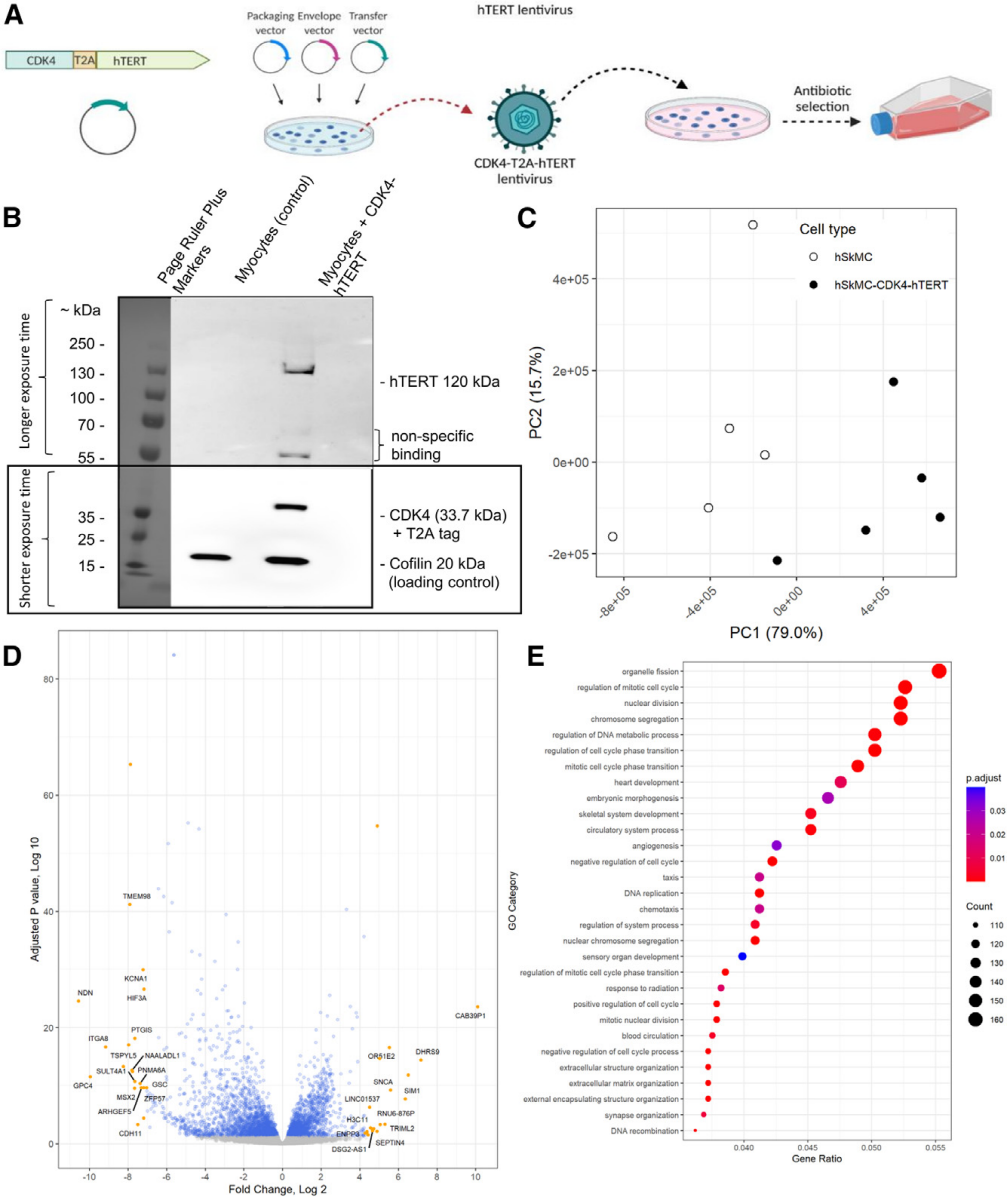
Production of immortalized muscle cells using lentivirus-mediated transduction of human primary myoblasts (A) Scheme for immortalization of muscle cells by lentivirus gene delivery. (B) Western blot detecting CDK4 and hTERT in myoblast lysates, with a cofilin loading control. Untransfected myocytes are used as a negative control. The blot in (B) was captured with two different exposure times to prevent over- or underexposure of the different bands. (C) PCA plot showing the first two PCs of the primary and immortalized muscle dataset. (D) Volcano plot showing the log_2_FC and the corresponding adjusted *p* values for each gene in the immortalized muscle cells with respect to the primary muscle cells. The values were calculated using the DESeq2 Wald method. The significant genes are indicated in blue and the not-significant genes are indicated in gray. Genes with particularly high log_2_FC are indicated in orange and labeled with the corresponding HUGO Gene Nomenclature Committee symbol. (E) Dot plot showing the top 30 GO terms. The color of the dots represents the adjusted *p* values, and the dot size is representative of the number of DEGs associated with the GO term. Benjamini-Hochberg used to adjust *p* value.

To facilitate the *in vitro* study of VEEV saRNA replication in human muscle cells, we generated a population of immortalized human myoblasts. We did this by transduction of primary cells with lentivirus-delivered fusion genes of *CDK4-T2A-hTERT* (Figure 1A). In primary myoblasts, progressive shortening of telomeres and activation of the p-16 ^INK4a^-mediated stress pathway are the two major contributors to cellular senescence. CDK4 has been shown to overcome senescence in myoblasts by blocking the p16 ^INK4a^-dependent stress pathway^11^^,^^12^; hTERT maintains telomere length, and over-expression is associated with cell immortalization in fibroblasts and endothelial cells. The T2A sequence was included because it causes the ribosome to skip the formation of a glycyl-prolyl peptide bond, resulting in two separate proteins from the same mRNA.^16^ Transduced cells were selected, with puromycin and expression of CDK4 and hTERT confirmed by western blot (Figure 1B). The immortalized muscle cells retained a similar morphology to primary muscle cells but grew more rapidly. While the primary cells typically stopped dividing after 10 passages due to cellular senescence, we were able to grow the immortalized cells for 30 passages. Once 30 passages were complete, we disposed of the cells; however, this is unlikely to be the limit of their capacity for cell division.

To validate the immortalized muscle cells further, we used transcriptomic profiling to compare the immortalized muscle cells to the original primary muscle population. Principal-component analysis (PCA; Figure 1C) indicates that the primary and immortalized muscle cells separate loosely across PC1 and PC2, but they do not form distinct clusters. Following PCA, we looked at the overall correlation in expression between the primary and immortalized muscle cells using Kendall’s rank correlation on all mean normalized gene counts. This gave an *R* value of 0.808 (*p* < 2.2 × 10^−16^), indicating a strong correlation between the baseline expression of primary and immortalized muscle cells. However, several genes were expressed very differently between the primary and immortalized muscle cells; therefore, we performed differential gene expression analysis with DESeq2 to investigate these genes.^17^^,^^18^ Out of 34,827 genes after filtering, we found that 4.5% of genes were upregulated and 6.4% were downregulated in the immortalized cells compared to the primary cells (Figure 1D; adjusted *p* < 0.05).

In addition to differential gene expression analysis, we used Gene Ontology (GO) analysis with clusterProfiler to investigate which biological processes the differentially expressed genes (DEGs) were involved in. The top 30 most altered GO categories between the primary and immortalized cells cluster into four distinct groups, specifically, (1) terms relating to chromosome segregation, (2) cell-cycle, (3) DNA replication, and (4) extracellular matrix organization (Figure 1E). These terms reflect the insertion of *CDK4* and *hTERT* genes and corresponding changes in the expression of genes associated with nuclear division, cell division, and the cell cycle. This also supports the observation that immortalized cells grow faster and to a higher passage than -primary cells. We did not see any statistically significant differences in immune- or inflammation-related GO terms. Overall, this analysis provides confidence that the immortalized muscle cells can be used as a convenient model to study RNA vaccine expression in human muscle.

### Transfection and expression of GFP from saRNA is associated with upregulation of the host transcriptome

**Figure 2:**
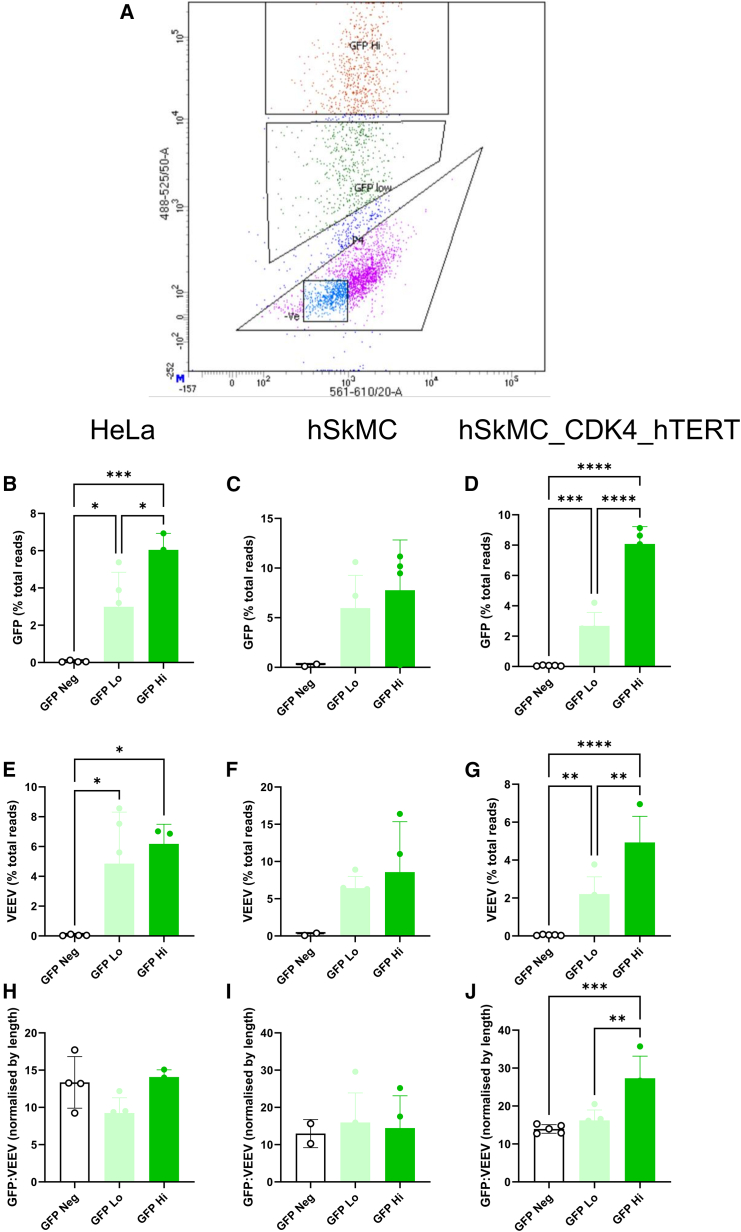
Uptake of saRNA and antigen expression is associated with increased host gene expression in primary and immortalized muscle cell lines Cells were transfected with 1 ng/μL VEEV GFP (total amount 1 μg) on 10^6^ cells, 16 h after transfection. (A) Cells were sorted for GFP expression, splitting into the top 40% brightest (GFP Hi), bottom 40% (GFP Low), and GFP^−^ (-ve). GFP subgenomic mRNA counts (B–D) and VEEV genomic replicon (E–G) counts were calculated as a percentage of total reads in sorted cells by cell type: HeLa (B and E), hSkMC (C and F), and hSkMC_CDK4_hTERT (D and G). GFP to VEEV ratio normalized to length (H–J). ∗ *p* < 0.05; ∗∗ *p* < 0.01; ∗∗∗ *p* < 0.001; ∗∗∗∗ *p* < 0.0001.

Having generated the immortalized human muscle cells, we used them alongside the primary muscle cells and HeLa cells to characterize the human muscle transcriptomic response to VEEV saRNA. We transfected human cells with lipofectamine-formulated VEEV saRNA, encoding GFP as a reporter antigen for 16 h. Lipofectamine was chosen because it gave a higher expression level than polymer-based formulations (polyethyleneimine [PEI] and poly[CBA-*co*-4-amino-1-butanol] [pABOL]) (Figures S1A and S1B). The RNA dose was chosen as 1 ng/μL (1 μg total); doses above this reduced transfection, possibly because of the inhibitory effects of RNA sensing (Figures S1C and S1D). A time point of 16 h post-transfection was chosen to optimize the percentage of GFP^+^ cells available for sorting and to maximize RNA yield for library synthesis from cells after sorting (Figures S1E and S1F). Cells were fluorescence-activated cell sorting (FACS) sorted based on their GFP expression levels into high, low, and negative (Figure 2A) before RNA extraction for RNA-seq analysis.

There was a sharp peak in GC content at 58%. This was only seen in the GFP-expressing cells. To determine whether GFP mRNA could be detected, the GFP transcripts were measured as a percentage of unassigned reads; in all three cell lines there were no detectable GFP reads in mock or untreated cells, but GFP could be detected in the transfected cells, so we focused our analysis on GFP as a proportion only in the sorted, treated cells. GFP was calculated as a percentage of the total reads (mapped and unmapped). GFP^−^ had some detectable GFP reads, but they were significantly lower than the higher-expressing cells (Figures 2B–2D). In the GFP high cells, the GFP accounted for between 5% and 10% of all the RNA reads. It was also possible to detect VEEV replicon RNA with a similar pattern—cells with higher GFP mean fluorescence intensity (MFI) had more VEEV (Figures 2E–2G); this accounted for a further 5%–10% of RNA reads. If replicating, the replicon makes more copies of the subgenomic gene of interest than the whole construct. We also looked at subgenomic GFP levels compared to genomic VEEV. Levels were normalized by RNA length to account for the longer VEEV. We observed an enrichment of GFP relative to VEEV, suggesting that the subgenomic RNA is being replicated. In the HeLa and hSKMC cell lines, there was no difference in the ratio between VEEV and GFP, indicating that the replication was constant regardless of expression level.

**Figure 3:**
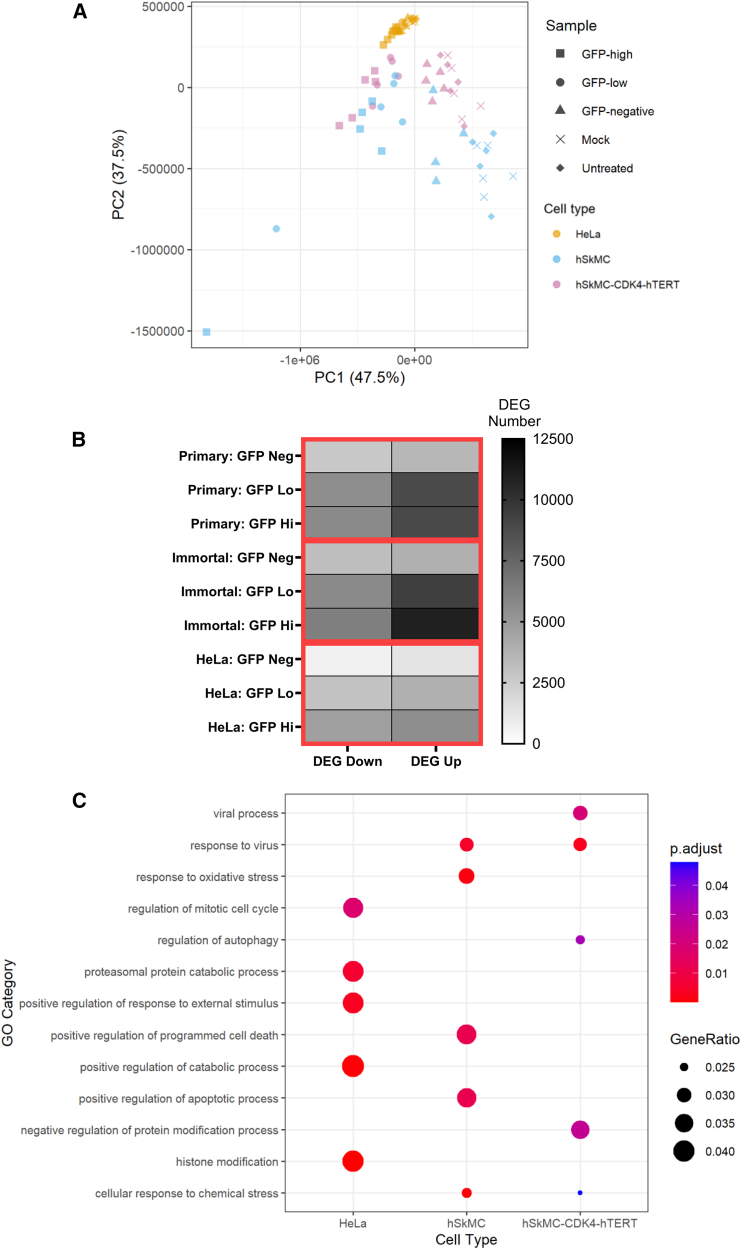
Uptake of saRNA and antigen expression are associated with increased host gene expression in primary and immortalized muscle cell lines (A) After sorting, RNA was extracted and global responses in different treatments/cell types were assessed by PCA. (B) Differential gene expression was determined with DESeq2; overall numbers of DEGs for each condition with an adjusted *p* < 0.05 cutoff (Benjamini-Hochberg). (C) DEG were clustered by GO term. *N* = 3 replicates per cell type/condition.

Having seen a difference in the GFP RNA levels when cells were sorted by expression level, we then explored whether there was also a difference in the host response. PCA was performed to visualize the variation between samples (Figure 3A). The HeLa samples formed a distinct cluster from the muscle samples, with little difference in the HeLa samples between the transfected and untransfected samples. There was some separation between the GFP-expressing muscle samples from the negative/mock and untreated samples. We compared the transfected samples (GFP^−^ and GFP high) to the untransfected baseline samples using the Wald method from the DESeq2 package. Overall, there was an increase in DEGs with increased GFP expression, this was seen in all cell types (Figure 3B). However, for all cell types and conditions the majority of genes (>60%) were not significantly differentially expressed. We also confirmed that there were not any statistically significant DEGs in the mock transfected samples compared to the untransfected samples, showing that any differential expression in the transfected samples can be attributed to the saRNA. Cluster profiler analysis was performed for the most significant genes identified by the Wald test across all conditions compared to the untransfected sample. There were differences in GO terms for the different cell types, with more genes in terms relating to the cell cycle in the HeLa cells and more in antiviral GO terms in the muscle cells (Figure 3C).

**Figure 4:**
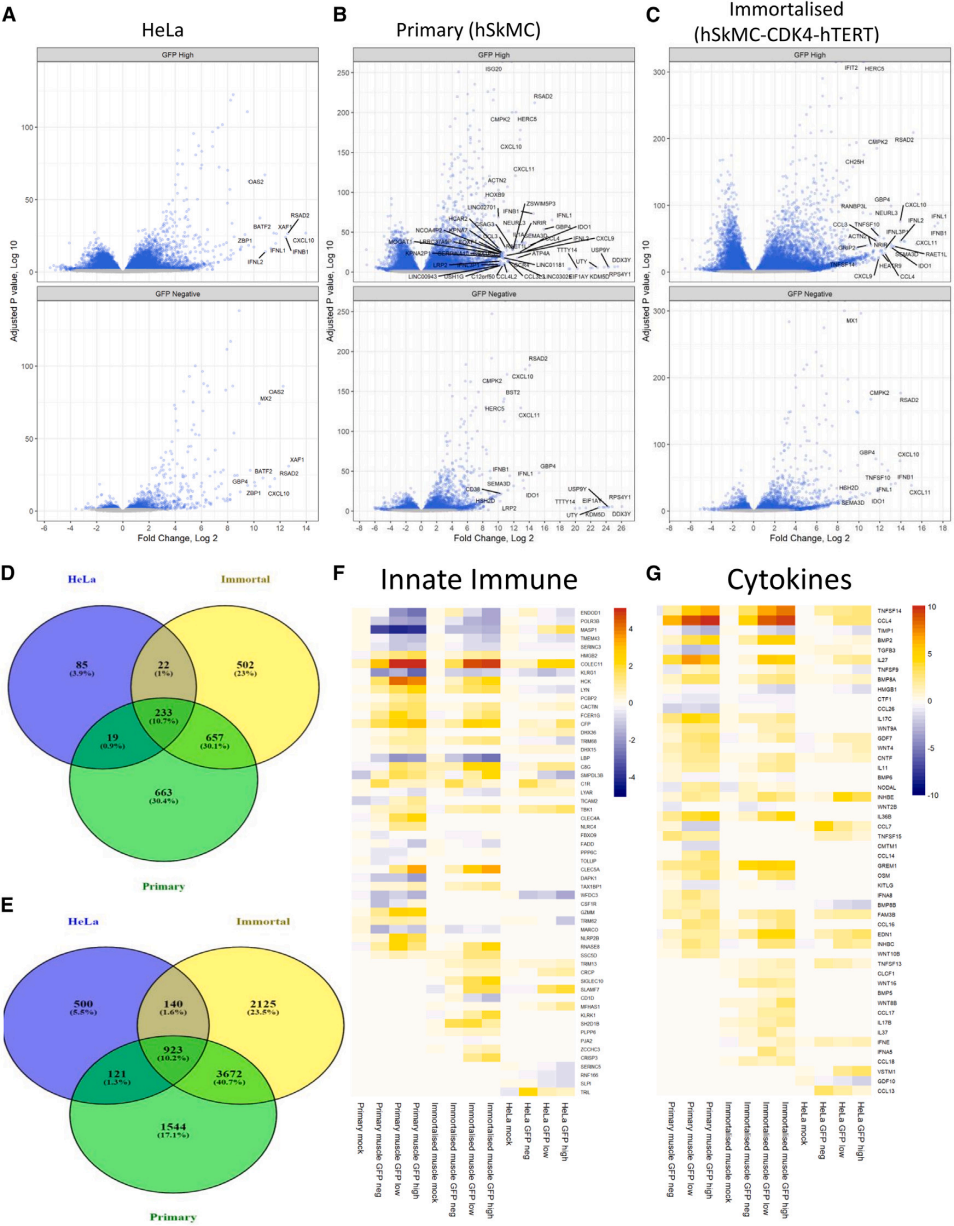
Cells with high GFP expression following transfection with VEEV upregulate similar patterns of innate immune genes Cells were saRNA transfected, sorted for GFP expression, and RNA-seq analysis performed. (A–C) Differential gene expression between transfected and mock control cells for (A) primary muscle cells, (B) immortalized muscle cells, and (C) HeLa cells. (D and E) Venn diagram of shared genes between GFP^−^ (D) and GFP high (E) cells, with FC >2 and *p* < 0.05 cutoff. (F and G) Heatmap of expression of significant (*p* < 0.05) differentially expressed innate immune (F; GO: 0045087) and cytokine activity (G; GO: 0005125) genes. Tile color represents log_2_FC from untreated control samples, and genes are clustered based on the similarity of expression profile. *N* = 3 replicates per cell type/condition.

Having seen increased gene expression with GFP transgene expression in all cell types, we initially looked at similarities between the GFP high expressors for the three different cell types. Differential gene expression analysis was visualized by volcano plot, reflecting the overall numbers of DEGs; there were fewer significantly different genes in the transfected HeLa cells compared to untransfected cells (Figure 4A). For both primary (Figure 4B) and immortalized (Figure 4C) muscle cells, there were more significantly upregulated genes in the GFP high cells than the GFP^−^ cells.

While the volcano plots show that the muscle cells have a greater number of highly upregulated genes than the HeLa cells, we can also see many of the same genes within the most highly upregulated genes in all three cell types. We compared the DEGs with a greater than 2-fold change between the GFP^−^ expressors for each cell type (Figure 4D). The overlapping genes included *MX1*, *OAS2*, *IFIT1*, and *ZC3HAV1* (encoding ZAP), all of which have been shown to restrict alphaviruses.^19^ There were also many overlapping genes between the different cell systems in the GFP high populations (Figure 4E), including *MX1*, *OAS2*, and *IFIT1*.

To investigate the DEGs further, we used the LRT (likelihood ratio test) option in DESeq2 to investigate the change in gene expression across the different levels of the samples in the order untransfected, mock, GFP^−^, GFP low, and GFP high. We created heatmaps to look at the overall expression patterns of genes identified as significant within the innate immunity (Figure 4F; GO: 0045087) and cytokine activity (Figure 4G; GO:00051251) GO terms, focusing on the top 20 most significant genes identified by LRT within a GO term. There were clear overlaps in both the innate immune and cytokine activity responses between the different cell lines and conditions. This was especially apparent for the primary and immortalized muscle cells, which clearly have a very similar innate immune response to the VEEV saRNA. Analysis of the DNA transcription factor activity GO term (GO: 0003700) indicated that six genes are common between all three cell types: *PAX5*, *ATF3*, *IRF1*, *ETV7*, *IRF7* (IFN regulatory factor 7), and *BATF2*.

### Comparison of response between GFP high and GFP^−^ cells as a way to identify potential restrictive factors

**Figure 5:**
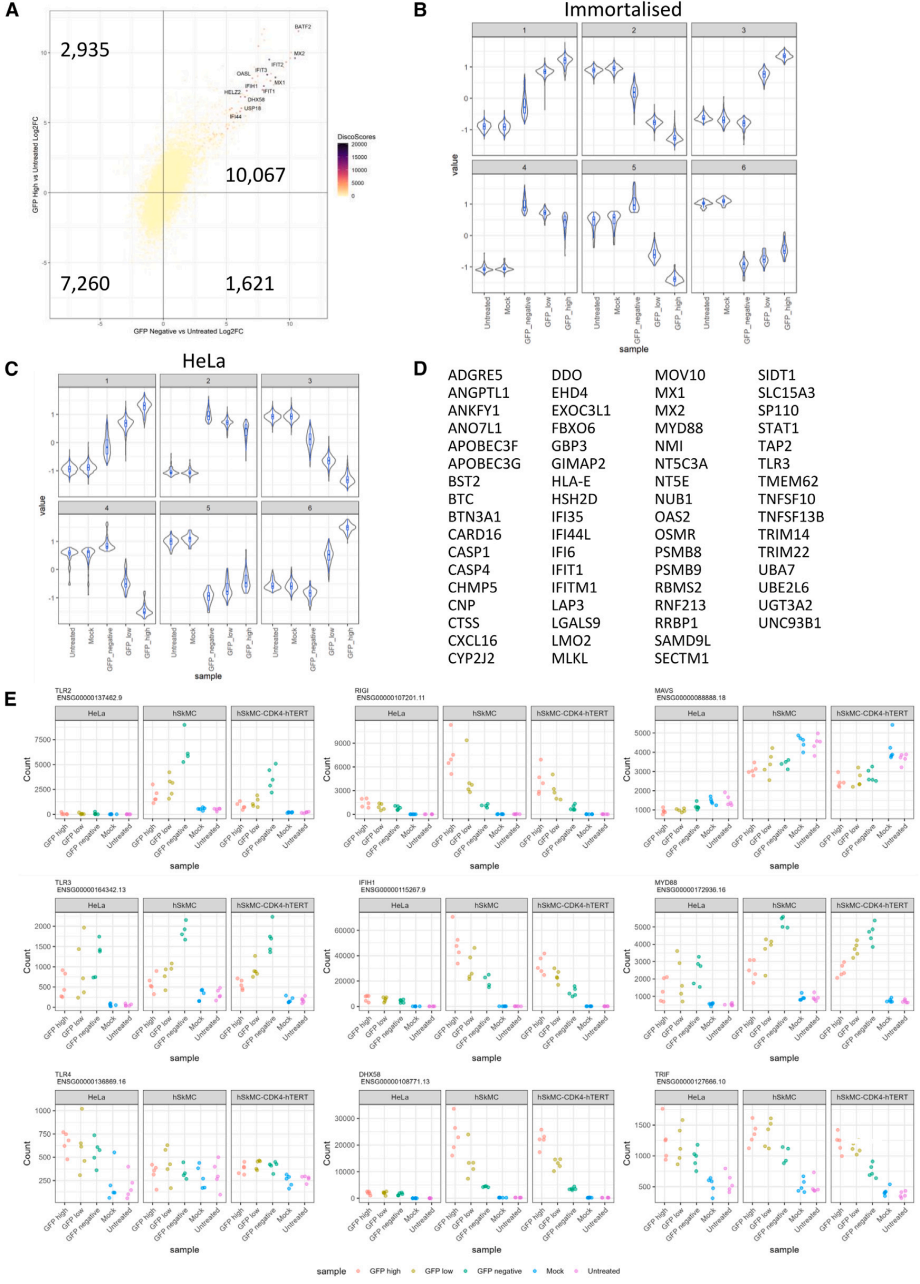
GFP high and GFP^−^ cells had differences in host gene expression associated with the innate immune response (A) Concordance and discordance (using the DISCO tool) between HeLa GFP high and GFP^−^ cells. (B and C) Comparing the differences between GFP-high and GFP^−^ RNA-seq datasets, genes were clustered using the degPatterns tool for immortalized muscle (B) and HeLa (C) cells. (D) Individual genes in all three clusters. (E) Expression (normalized counts) of individual genes of interest.

One aim of the study was to determine whether there were differences in host cell gene responses between cells with high and no transgene expression that may restrict the function of the saRNA in target cells. We determined whether there were global differences in the RNA transcriptome between GFP high and GFP^−^ cells. To do this, we assessed the concordance and discordance between GFP high and GFP^−^ cells using DISCO for immortal cells (Figure 5A). DISCO identifies whether there are genes that were differentially upregulated or downregulated between the two conditions compared to the untreated baseline by comparing significant DEGs identified in the Wald test. Of the DEGs, 10,067 were upregulated in both GFP high and GFP^−^ cells, 1,621 were upregulated in GFP^−^ but not GFP high cells, and 2,935 were upregulated in GFP high but not GFP^−^ cells. This indicates that rather than different profiles, differences in transgene expression might be a result of different levels of host gene transcription. We hypothesized that the expression of restrictive genes would be higher in the GFP^−^ cells than the GFP^+^ cells. To identify these genes, we used the degPatterns tool as an unsupervised approach to cluster genes according to the overall expression pattern across each of the samples from the untreated samples, GFP^−^, GFP low to GFP high samples. Focusing on the HeLa and immortalized muscle cells, we performed this using the top 5,000 most significant DEGs from each cell type identified with the LRT. Clusters 4 and 5 for the immortalized muscle cells (Figure 5B) and clusters 2 and 4 for the HeLa cells (Figure 5C) fit the criteria for containing potentially restrictive genes, where the expression is highest in the GFP^−^ cells. To investigate the functionality that these candidate genes may have, we performed GO analysis with clusterProfiler. The top 30 GO terms that were significantly (adjusted *p* < 0.05) enriched in the clusters of interest all related to the innate immune response, especially the antiviral response. We drilled further down into the clusters to identify individual genes associated with the restrictive effect (Figure 5D). Genes that were higher in negative cells included *IFIT1*, which is thought to inhibit alphavirus replication and translation initiation,^20^ and the restriction factors *MX1* and *TLR3*, associated with sensing RNA.

Since many of the genes that were more upregulated in the GFP^−^ cells were ISGs and associated with the innate immune response, we investigated whether there were differences in genes associated with sensing and responding to RNA. In the GFP^−^ muscle cells, there was greater expression of *TLR2* and *TLR3* compared to GFP high cells (Figure 5E); there was no difference in *TLR4*. However, the cytosolic RNA receptors *DDX58* (RIG-I), *IFIH1* (MDA5), and *DHX58* (LGP2) were higher in the GFP high cells than in the GFP^−^ cells; there was no difference in the gene *MAVS* that encodes the signaling molecule that interacts with these proteins. There was a greater level of *MYD88*, the protein that interacts with TLR2 in GFP^−^ cells compared to GFP high, but *TRIF* (which encodes a protein that interacts with TLR3) was higher in GFP high cells. These findings suggest that cells transfected with saRNA that do not express GFP have a higher level of a subset of ISGs that could block expression of the saRNA.

### The effect of blocking IFN signaling with ruxolitinib treatment

**Figure 6:**
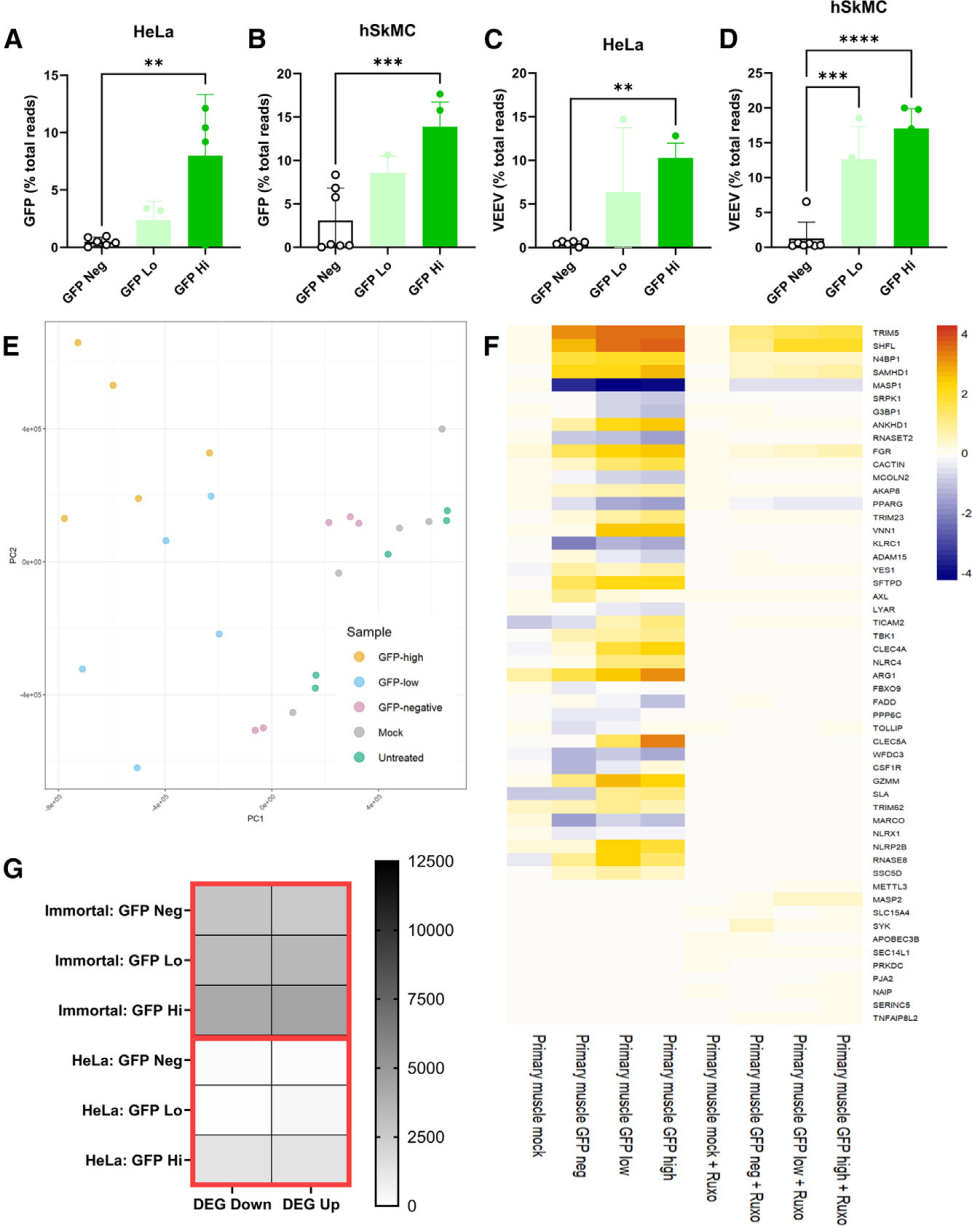
The JAK-STAT inhibitor (ruxolitinib) reduces the number of DEGs following saRNA transfection, but there were still differences between cells when sorted by expression level HeLa and primary muscle cell line were treated with 30 ng/μL ruxolitinib (Ruxo) before transfection. Cells were transfected with 1 ng/μL VEEV GFP; 16 h after transfection, cells were sorted for GFP expression, splitting into the top 40% brightest (GFP Hi), bottom 40% (GFP Lo), and GFP^−^ (GFP Neg). After sorting, RNA was extracted for RNA-seq analysis. GFP subgenomic mRNA counts (A and C) and VEEV genomic replicon (B and D) counts were calculated as a percentage of total reads in sorted cells by cell type: HeLa (A and C) and hSkMC_CDK4_hTERT (B and D). (E) Global responses in different treatments/cell types were assessed by PCA. (F) Heatmap of expression of significant (adjusted *p* < 0.05, Benjamini-Hochberg) differentially expressed innate immune genes (GO: 0045087). (G) Differential gene expression between transfected and mock control cells for immortalized muscle cells. ∗ *p* < 0.05; ∗∗ *p* < 0.01; ∗∗∗ *p* < 0.001; ∗∗∗∗ *p* < 0.0001.

It was interesting that the GFP^−^ cells showed strong upregulation of many of the same genes as the GFP^+^ sample, despite showing no antigen expression. This could be due to IFN signaling from neighboring cells that had taken up the saRNA and responded to it; whether this is other negative cells or the GFP-expressing ones is not known. To differentiate direct innate sensing of saRNA from the paracrine effects of induced IFNs, HeLa or primary muscle cells were treated with ruxolitinib during transfection to prevent JAK1/2 signaling of the IFN-α/β receptor. We compared responses with and without ruxolitinib. We saw no change in the number of GFP^+^ cells after ruxolitinib treatment in human cell lines (Figure S3A), but there was a significant increase in the MFI of GFP (Figure S3B); we also saw a significant increase in expression using a different readout (luciferase; Figure S3C). There was still a gradient in GFP mRNA from high to low expressing cells (Figures 6A and 6B), but the percentage of total reads was higher than the untreated cells; there were also more detectable reads in the negative cells than seen in the untreated cells. A similar pattern was observed with the VEEV (Figures 6C and 6D). In the hSkMC cells, the VEEV reached 20% of all RNA reads; when combined with GFP, the transgene accounted for nearly 35% of all the RNA in the cell.

To assess whether ruxolitinib altered the transcriptome, we initially compared global patterns in gene expression between all the ruxolitinib-treated cells by PCA (Figure 6E). Within the muscle cells, there was some directional clustering with GFP high cells furthest away from mock and untreated cells and GFP^−^ cells clustering with mock cells. In comparison to the results from the samples without ruxolitinib, treated samples have far fewer significant DEGs (Figure 6G). In the HeLa cells, there were very few DEG, so we focused on immortalized muscle cells. Again, we confirmed that there were no statistically significant DEGs in the mock transfected samples compared to the untransfected samples.

A heatmap of innate immune response genes in primary muscle cells (GO: 0045087; Figure 6F) demonstrates a clear reduction in the response to saRNA in the ruxolitinib-treated samples, with fewer genes showing up- or downregulation and a reduction in the size of the change in expression of these genes. Despite the presence of a JAK-STAT inhibitor blocking potential IFN signaling, upregulation of innate immune genes was clearly detected in the GFP^−^ cells. To investigate this further, we used the DESeq2 Wald test to directly compare the change in gene expression between the control untransfected and the GFP^−^ samples. This identified 50 significant innate immune-related DEGs in the primary muscle GFP^−^ cells. These results show that despite ruxolitinib treatment, there is still significant upregulation of innate immune genes in the GFP^−^ cells. Out of the six upregulated transcription factors common to all the cells without ruxolitinib, only *BATF2*, *IRF7*, and *ETV7* remain strongly upregulated in the ruxolitinib-treated samples.

### Comparison of mouse and human responses to VEEV saRNA

The muscle is the main site of immunization for RNA vaccines, but it is difficult to access human muscle directly after immunization, which is where *in vivo* mouse studies can be beneficial. However, there are differences in the response between species, and having bridging data could help predict how changes to saRNA constructs can affect the innate immune response and potentially affect expression and immunogenicity. To understand differences between human and murine muscles cells, we performed RNA-seq on mouse C2C12 myoblasts. This cell line was used because we had also used an immortalized human muscle cell line for the analysis. Because of the surmised impact of paracrine IFN signaling on GFP^−^ cells in the human cell system, we compared responses in ruxolitinib-treated mouse cells. As before, cells were transfected with VEEV GFP in the presence of ruxolitinib and sorted into GFP^−^, GFP low, and GFP high groups following a 16-h incubation.

**Figure 7:**
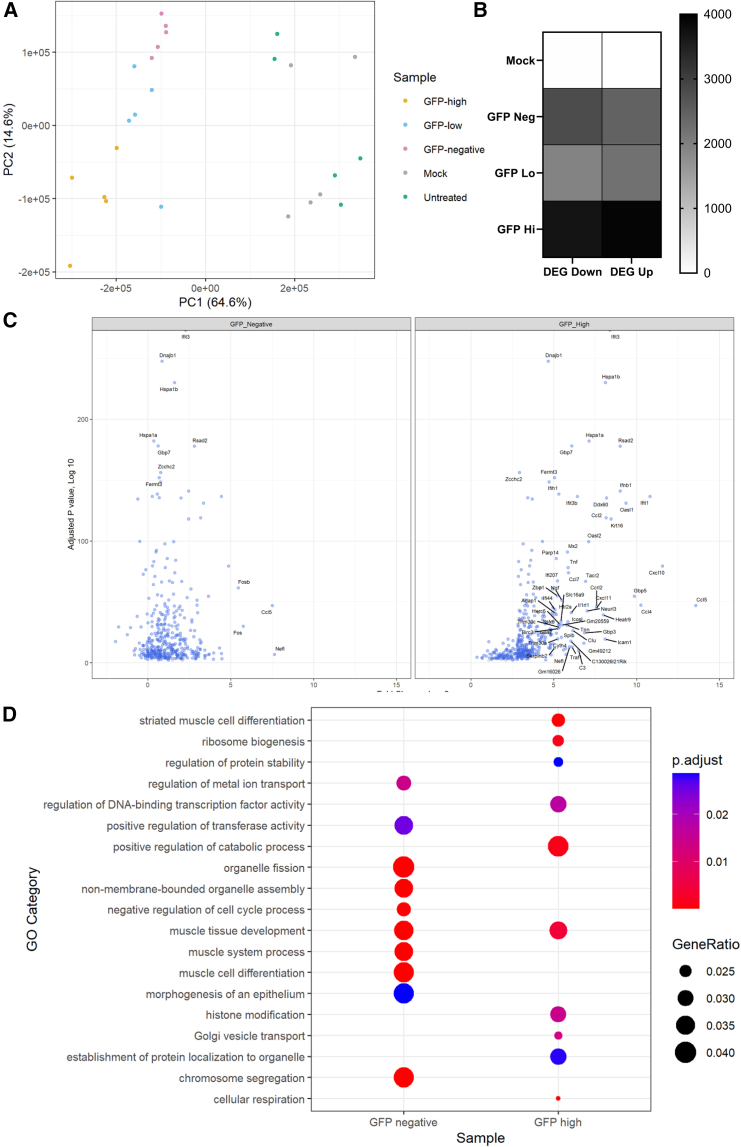
Transcriptomic responses to saRNA in mouse muscle Mouse C2C12 myoblast cells were transfected with 1 ng/μL VEEV GFP in the presence of 30 ng/μL ruxolitinib. At 16 h after transfection, cells were sorted for GFP expression, splitting into the top 40% brightest (GFP Hi), bottom 40% (GFP Lo), and GFP^−^ (GFP Neg). (A) After sorting, RNA was extracted, and global responses in different treatments/cell types were assessed by PCA. (B) Differential gene expression was determined by the LRT function of DESeq2; overall numbers of DEG for each condition with an adjusted *p* < 0.01 cutoff (Benjamini-Hochberg). (C) Differential gene expression between transfected and mock control cells. (D) DEGs were clustered by GO term. *N* = 3 replicates per cell type/condition.

We performed PCA to look at global patterns of host gene expression after transfection with VEEV. The different GFP-expressing groups separated on the PCA plot, with GFP-expressing cells clearly separate from the mock or untreated cells (Figure 7A). In the GFP high population, there were 3,911 upregulated DEGs (Figure 7B). Volcano plot analysis of DEGs shows that there were significantly fewer upregulated genes in the GFP^−^ (Figure 7C) than the GFP high (Figure 7D) cells. As seen with the human cells, the DEG clustered GO terms associated with muscle cell differentiation and catabolism (Figure 7E). There were no DEGs identified between the mock transfected and untreated samples.

We then compared responses to human cells. One observation was that the fold change of RNA was greater in the mouse cells than in the human cells. An example of this is in the fold change of chemokine genes (GO: 0008009; Figures 8A and 8B). There was a similar panel of upregulated chemokine gene expression, and human cells significantly upregulated a total of 26 chemokines compared to 18 for the mouse muscle cells (adjusted *p* < 0.05). However, the mean maximum log_2_fold change (FC) across all samples for the mouse chemokines was 4.98, compared to only 0.67 for the human chemokines. Whether this translates into more protein is not clear.

**Figure 8:**
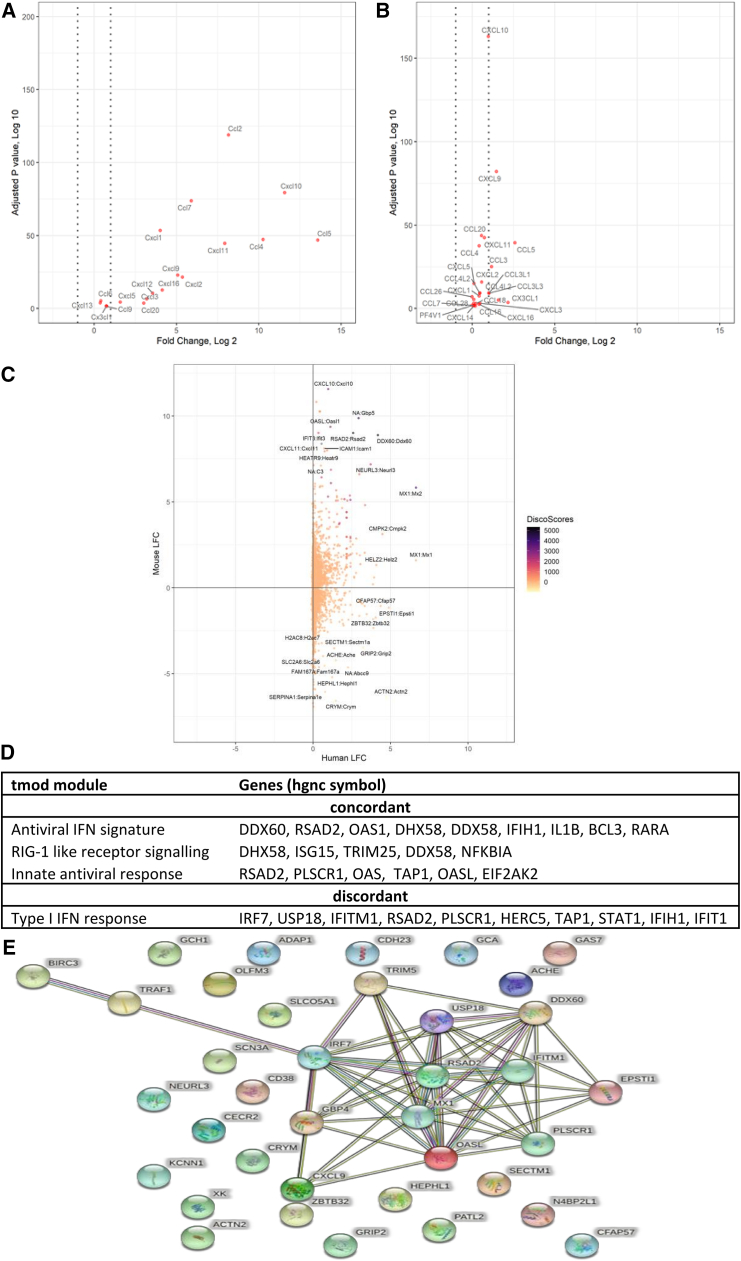
Both human and mouse cell lines express IFN-related genes in response to saRNA, but there are differences between the cell lines RNA-seq data from the human and mouse myoblast cell lines were compared. (A and B) Log_2_FC in expression across all samples (mock, GFP^−^, GFP low, GFP high) compared to the baseline untreated sample for chemokine activity (GO: 0008009) in mouse (A) and human (B) myoblasts. (C) DISCO analysis of top 400 human DEGs compared to mouse orthologs. (D) Key concordant and discordant gene sets. (E) STRING analysis of most discordant genes by expression level in human cells.

There are some differences in gene names between the species to allow for comparison of the murine and human transcriptomic response. We used the BiomaRt getLDS function, which uses the Ensembl database to pair orthologous genes in mice and humans. To investigate the differences between murine and human muscle responses to VEEV saRNA more directly, we used the DISCO tool (Figure 8C).^21^ We compared the top 400 significant DEGs (determined by maximum absolute log_2_FC across all samples compared to the untreated sample) from the ruxolitinib-treated primary human muscle samples to the orthologous murine genes. For the top 400 differentially expressed human genes, there were 225 equivalent murine genes, and of these, 137 were also differentially expressed in the murine muscle cells. We then applied gene set enrichment analysis to the concordant and discordant gene lists using the R package tmod. tmod identified 27 sets of concordantly expressed gene sets, almost all of which were innate immune related, including antiviral IFN signature, RIG-1-like receptor signaling, and innate antiviral response (Figure 8D). A total of 57 sets of genes were also found to be significantly enriched in the discordant gene list, including type I IFN response. To investigate the discordant genes further, we created a list of genes that were both highly discordant (greater than a 10-fold difference in expression) and upregulated highly in humans over mice and used these genes as an input to the STRING database (Figure 8E). This indicates a set of interacting proteins that are centered on the transcription factor IRF7, indicating that the expression level of *IRF7* could have a large impact on the activity of the other proteins in humans.

## Discussion

In this study, we investigated the cell-intrinsic response to self-amplifying RNA vaccines, developing a set of immortalized human muscle cells to facilitate our experiments. We showed that the transfection of primary human myoblasts with hTERT and CDK4 led to immortalization of primary human myoblast cells. We then used these cells to explore the cell-intrinsic response to VEEV saRNA encoding GFP. To explore the interplay of transgene expression and host cell response, we sorted cells based on GFP level. We observed a significant upregulation of host genes following transfection in cells regardless of expression level. To rule out the effect of paracrine IFN, we treated cells with the JAK-STAT inhibitor ruxolitinib. We saw a significant reduction in the total number of significant DEGs in ruxolitinib-treated cells. However there were still differences between the cells sorted on the basis of GFP expression level.

Use of hTERT and CDK4 to immortalize muscle cells has been used previously to create a variety of cell lines, especially for the study of muscle-related diseases. This was the first time a T2A sequence was used to express both proteins from a single transgene. These techniques are popular because primary human muscle cells expand slowly and have a very limited proliferative capacity, which can make them challenging to work with. The process of immortalizing the myoblasts results in cells that divide much more quickly and for longer before senescence occurs, an effect that we also observed with our immortalized population. Other studies have isolated individual cells to generate homogeneous clonal populations of cells^11^^,^^12^; however, we chose to retain a more heterogeneous population to better mimic the phenotypic diversity within a primary cell population. While we would expect some transcriptional changes to occur as part of the immortalization process, we wanted the immortalized population to be as representative of the primary population as possible. The correlation analysis and differential gene expression analysis suggest that the immortalized and primary muscle cells are very similar in terms of baseline transcription.

Having generated the immortalized cell line, the aim of the study was to understand whether there were underlying differences in cells that do or do not express GFP following transfection with saRNA. This was to identify potential cell-intrinsic blocks to expression as a way to understand the expression dynamics of saRNA and ultimately use this to improve the vaccine platform. The *a priori* hypothesis was that GFP^−^ cells would have greater upregulation of genes such as IFNs or ISGs that inhibit expression of the saRNA transgene. Heterogeneity in cellular responses to viruses has previously been observed for viral infections^22^; since the saRNA construct we use is virally derived (based on VEEV), there are likely to be heterogeneous responses. An equivalent calculation to MOI for RNA is challenging, but we added 1 μg to 10^6^ cells; this works out to be about 20 RNA-containing particles per cell. It has been estimated that only 1% of this material then escapes the endosome into the cell.^23^ There will be some uneven distribution of uptake and expression, even in the absence of other factors; we have not included an uptake marker, such as labeling the RNA to address this. An important question is how this then translates from a single-cell system into the much more complex anatomy following injection into a human muscle.

Our data demonstrate that GFP is an extremely highly expressed gene in these cells. When combined with the VEEV RNA reads in the ruxolitinib-treated cells, it accounted for nearly 35% of all reads. This echoes research that the subgenomic transcript from alphaviruses becomes the most abundant RNA in the target cell.^24^ Understanding the impact of this on the host cell and then how that affects the adaptive immune response will be important. In addition to the impact of RNA, the encoded protein may stress the cell when expressed at high levels; for example, GFP can lead to oxidative stress in the cells.^25^ This may also alter the downstream transcriptional response.

We then sought to understand why there might be different levels of GFP RNA in cells expressing different levels of protein. It is worth noting that we observed different patterns of host responses between cells that did and did not express GFP. The main differences observed were in genes clustered in GO terms relating to antiviral immunity; with a number of ISGs that might inhibit saRNA replication or expression, such as *IFIT1* and *MX1*. IFIT1 has recently been shown to be important in restricting expression by VEEV-GFP^19^; however, it should be noted that VEEV has evolved a 5′ UTR structure that can evade IFIT1.^26^^,^^27^ There was also an increase in some immune-sensing genes. Both *TLR2* and *TLR3* are upregulated to a greater extent in the GFP^−^ cells than the GFP^+^ cells, which is intriguing as TLR3 is a double-stranded RNA (dsRNA) sensor that is only normally constitutively expressed in professional APCs. This greater capacity to sense dsRNA, such as the saRNA double-stranded replication intermediate, could contribute to the lack of antigen expression in GFP^−^ cells. However, the cytosolic RNA sensors *RIGI*, *IFIH1* (encoding MDA5), and *DHX58*, which have previously been associated with the sensing of RNA,^28^ were higher in the GFP high cells.

RNA sensing is critical in the response to RNA vaccines. Much of the success of mRNA vaccines has been attributed to replacing the uridine with pseudouridine, thereby reducing the type I IFN response.^29^ We have shown that this alteration leads to a significant alteration in the gene profile in lymph nodes after immunization.^30^ Therefore, it was of interest that in the individual cell system there was evidence of RNA sensing and higher levels of some cytokines such as *CCL4* and *IL27* in the GFP high cells. There were overlaps in transcription factors between the cell types that were associated with IFN sensing. For example, IRF1 is known to induce the expression of antiviral genes in response to many different viruses, including encephalomyocarditis virus,^31^ West Nile virus,^32^ and murine norovirus,^33^ and has specifically been shown to have a limiting effect on Chikungunya^34^ infection in muscle cells.

Since we saw the upregulation of cytokine genes from GFP high cells, we speculated that signaling from one cell to another may have a paracrine, *trans*-acting effect with cells that take up the RNA early, inhibiting the responses in others. To investigate this, we used the JAK/STAT inhibitor ruxolitinib. Blakney et al. had previously shown that treatment with ruxolitinib improved expression of the antigen from VEEV saRNA.^35^ We also saw an increase in luciferase expression in our optimization studies. The ruxolitinib-treated cells had a different profile of responses, with no increase in GO terms relating to the innate response. One caveat of this analysis is that the cells were collected at different times, so it is not a direct comparison, but there were far fewer DEG in the ruxolitinib-treated cells. There was no upregulation of genes encoding key transcription factors IRF1 and ATF3, which are known to mediate the expression of antiviral and pro-inflammatory genes. This may help to explain the increase in GFP signal in ruxolitinib-treated cells and suggests that much of the gene upregulation following saRNA transfection is driven by signaling through JAK/STAT. Estimates vary about the number of IFN-responsive genes, but one study estimates there could be as many as 2,000 human genes affected by IFN,^36^ which could explain why the number of DEGs is so much lower. Overall, we observed that even in the absence of JAK-STAT signaling, the expression of saRNA encoded transgene was heterogeneous and that it still altered the host transcriptome.

How far observations in mouse models can be translated into human responses is something we wanted to address here. An underlying question is why mice respond uniformly well to saRNA vaccines (at least in some inbred strains) compared to humans, especially to a neoantigen. In the first human studies using this platform, 20% of baseline naive individuals did not seroconvert to an saRNA vaccine.^37^ We investigated whether there were differences between mouse and human muscle cells that might contribute to this. While patterns of gene expression were similar, the magnitude of the FC was far greater in mice. This is corroborated by the findings of Pepini et al., who used microarrays to measure changes in local gene expression following intramuscular injection with a self-amplifying RNA vaccine.^1^ They identified the upregulation of several chemokines, including *Ccl2*, *Ccl7*, *Cxcl9*, *Cxcl10*, and *Cxcl11*, all of which are also very significantly upregulated in our samples. Whether RNA transcript levels translate into protein in a uniform way in different species needs to be explored to draw conclusions on the impact of this, but it is notable that mice respond better to the vaccine and make more of the chemokines that might recruit cells to the site of immunization.

We set out to investigate factors that affect saRNA antigen expression at a cellular level as a way to understand factors that could reduce transgene translation. What was striking was that the cells that make antigens also make many of the genes we might predict to be inhibitory. The exact kinetics of transgene expression vs. host response are not captured here based on bulk sequencing rather than individual cell data. How this affects the immunogenicity of the saRNA vaccine platform needs further evaluation. While the first-in-human trials were not immunogenic in all individuals,^37^ a large amount of further optimization is still required. There is still more to be understood about this complex process and how transgene translation translates into immunogenicity.

## Materials and methods

### Cell culture

#### Maintenance of cell cultures

HeLa cells were obtained from American Type Culture Collection and were maintained at 37°C and 5% CO_2_ in DMEM supplemented with 10% fetal calf serum, 1% l-glutamine, and 1% penicillin/streptomycin (henceforth, cDMEM). C2C12 cells were kindly gifted by Dr. Kai Hu and were also maintained in cDMEM.

hSkMCs were purchased from Promocell and were grown in Skeletal Muscle Cell Growth Medium with Supplement Mix (PromoCell, C-23060). Immortalized hSkMCs were generated in-house and were maintained in the same manner.

All cell lines were sub-cultured before reaching 70% confluency and grown for a maximum of 30 passages, or 12 passages in the case of the primary cells. Unless specified otherwise, all cell culture reagents were purchased from Gibco.

### Generation of immortalized hSkMCs

#### Lentivirus vector production

The cDNA sequences of hTERT and CDK4 were obtained from the NCBI’s GenBank and were used to design a CDK4-T2A cleavage sequence-hTERT construct that was synthesized as a DNA string by GeneArt (Thermo Fisher Scientific).

The cDNA construct was cloned into lentivirus transfer plasmids (pLenti Puro DEST, Addgene plasmid 17452, sequence 212855) using Invitrogen Gateway technology. The lentivirus vector was produced by transfecting HEK293T cells with the transfer vector, a packaging plasmid (psPAX2, plasmid 12260, Addgene), and an envelope plasmid (pMD2.G, plasmid 12259, Addgene). After 48 h, growth medium was collected and filtered using a 0.45-μm filter. Virus-containing medium was stored in 0.5-mL aliquots at –80°C.

### *Lentivirus infection*

We resuspended 1.5 × 10^5^ hSkMCs in 1.5-mL lentivirus-containing media, and polybrene was added at 8 μg/mL. Cells were centrifuged at 400 RCF for 90 min at room temperature (RT). The supernatant was discarded, and the pelleted cells were washed once in 6 mL growth medium before plating in fresh myocyte growth medium without selection. At 48 h following infection, selection was performed using fresh growth medium with 0.5 μg/mL puromycin (Thermo Fisher Scientific), replaced every 2 days for a total of 7 days. The selected cells were returned to normal growth medium for normal culture and storage.

#### Western blot

Samples of 3 × 10^6^ muscle cells were washed with ice-cold PBS before vortexing for 5 min in ice-cold lysis buffer with protease inhibitors. Lysate was clarified by centrifugation at 13,000 RCF for 10 min at 4°C, and reducing agent was added before boiling at 70°C for 10 min. Reduced samples were run on a Bolt Plus Bis-Tris gel, 4%–12% (Thermo Fisher, NW04120BOX) at 200 V for 30 min in Bolt MOPS SDS Running Buffer (Thermo Fisher, B000102). A PageRuler Plus pre-stained protein ladder was included for reference (Thermo Fisher, 26619). The gel was transferred to a nitrocellulose membrane at 30 V for 1 h. The membrane was blocked in 5% nonfat milk in PBS with 0.05% Tween 20 for 1 h at RT before incubating with primary antibodies overnight at 4°C (rabbit anti-CDK4, Abcam, ab68266) at 1:1,000, rabbit anti-hTERT (Abcam, ab32020) at 1:1,000, rabbit anti-cofilin (Abcam, ab42824) at 1:1,000. The membrane was washed for 10 min in in 5% nonfat milk in PBS with 0.05% Tween 20 four times. A goat anti-rabbit immunoglobulin G H&L (horseradish peroxidase [HRP]) (Abcam, ab6721) was added at 1:30,000 in in 5% nonfat milk in PBS for 1 h at RT and washed 4 times for 10 min with 5% nonfat milk in PBS with 0.05% Tween 20. The membrane was stained using HRP developing reagent (Thermo Fisher, 34580) and imaged using the Biostep Chemiluminescence- and Fluorescence-Imager Celvin S with SnapAndGo software.

### RNA manipulation

#### *In vitro* transcribed RNA synthesis

Capped RNA was synthesized *in vitro* from a linear DNA template using the mMESSAGE mMACHINE (Invitrogen, AM1344) kit and purified using lithium chloride precipitation. The yield of RNA was determined using a nanodrop spectrophotometer and the quality assessed by denaturing agarose gel electrophoresis. Samples of RNA prepared with glyoxal sample buffer (Lonza, 50560) were run alongside Millennium RNA Markers (Invitrogen, AM715) on a 1% agarose gel made with ultrapure agarose (Thermo Fisher, 16500100) and 1× NorthernMax-Gly Gel Prep/Running Buffer (Thermo Fisher, AM8678). The gel was visualized with a GelDoc-IT2 Imager to confirm RNA size and to check for degradation.

#### Transfection of adherent cells with lipofectamine

Lipofectamine MessengerMAX reagent was combined with Opti-MEM Medium and incubated for 10 min at RT before combining with an equal volume of RNA diluted in Opti-MEM and incubating for a further 5 min at RT. The RNA-lipofectamine formulation was pipetted directly into the growth medium of adherent cells at a maximum of 70% confluency to achieve a final dose of 1 ng/μL.

#### Transfection with PEI

Log-phase cells were harvested, seeded into 6-well plates (1.4 × 10^6^ cells/well), and left to adhere overnight at 37°C. Growth medium was aspirated and replaced with transfection medium consisting of a 7-μg dose of RNA complexed with PEI at a ratio of 1:4 (PEI:RNA), diluted in DMEM (1% penicillin/streptomycin), and incubated at 37°C for 4 h. Transfection medium was carefully removed and replaced with cDMEM for 24 h before further analysis.

#### Transfection with pABOL

Transfection with pABOL was performed as described by Blakney et al.^38^

### Transfection and preparation of samples for FACS and transcriptomics

hSkMC (5 × 10^6^), immortalized hSkMC (5 × 10^6^), and HeLa cells (8 × 10^6^) were plated in a T75 flask with growth medium and allowed to adhere overnight. Cells were transfected with a 1-ng/μL dose of VEEV GFP RNA (12.5 μg total) using lipofectamine. Mock cells were treated with empty lipofectamine in Opti-MEM Medium.

After incubation for 16 h at 37°C, cells were processed for cell sorting. Wherever possible, samples were kept on ice, and ice-cold reagents were used to maintain cell viability. Cells were harvested using trypsin and transferred to sterile 15-mL Falcon tubes. Cells were pelleted by centrifugation at 300 RCF for 7 min at 4°C before washing with ice-cold Dulbecco’s PBS (DPBS) and pelleting again. Pellets were resuspended in 4 μL Zombie Far-Red Live/Dead stain in 400 μL DPBS and were incubated in the dark at 4°C for 30 min. Cells were washed with 2 mL DPBS 10% fetal bovine serum before finally resuspending in growth medium to a final volume of 5 × 10^5^ cells/mL. The samples were transferred to FACS tubes by passing through a cell strainer to remove cell clumps. Samples were kept on ice until sorting.

Controls of unstained cells and 50% live/50% dead cells were used alongside the samples. The live/dead control was created by heating untransfected cells at 80°C for 5 min before mixing at a 1:1 ratio with live untransfected cells and staining with live/dead stain in the same way as the samples.

#### FACS

Cells were sorted into three populations (GFP^−^; GFP^+^, low expression; and GFP^+^, high expression) using a Beckton Dickinson FACS Aria III fitted with a 100-μm nozzle. During sorting, cells were collected into 1.5-mL sterile Eppendorf tubes containing 200 μL growth media at 4°C to minimize cell death. Cells were pelleted by centrifugation at 300 RCF, 4°C for 10 min, and resuspended in 300 μL TRIzol, before storing at −70°C.

#### RNA extraction

RNA was extracted using the Direct-zol RNA Miniprep kit by Zymogen (Zymo Research, R2051) with the additional 15 min on-column DNA digestion. RNA was eluted into 30 μL nuclease-free water and stored at −70°C for up to 2 weeks before shipping on dry ice to Novogene for analysis.

#### RNA-seq

RNA-seq was performed by Novogene. Novogene confirmed the quality of each total RNA sample before preparing libraries using either the standard-input or the low-input method, where the total RNA concentration was below 200 ng/sample. Libraries were sequenced with paired-end 150 reads using the Illumina NovaSeq 6000. A minimum of 30 million reads per sample was generated. Novogene performed basic data quality control analysis.

### Data analysis

#### Pre-processing of sequencing data

Raw sequencing reads were trimmed with Trimmomatic (version 0.38) using the following operations: Illumina universal adapters were removed with ILLUMINACLIP; sliding window trimming was used to trim reads, with an average quality score of less than 15 across a window of 4 bases; and the data were filtered to remove reads less than 36 bases in length. The quality of trimmed reads was confirmed using FastQC (version 0.11.9), and reports were combined using MultiQC (version 0.6). Reads were aligned to the human reference genome GRChg38 or mouse reference genome GRCm39 with annotations from GENCODE using RNA STAR (version 2.7.3a) before count files were generated using HTseq (version 0.11.1).

#### Further analysis of sequencing data

All further data analysis was performed in R (version 4.3.1). Initial PCA analysis was performed on all normalized counts using the DEseq2 median of ratios method and the prcomp function from the R Stats package (version 4.3.1).

Differential gene expression analysis was performed using DESeq2 (version 1.40.2). Pre-filtering of the genes to remove low counts was not performed due to the built-in strict independent filtering step on the mean normalized counts automatically applied by the DESeq2 results function. Both the LRT and the Wald test were used for hypothesis testing, as specified in the test, with an adjusted *p* value threshold of 0.05. Where multiple testing correction was required, the Benjamini-Hochberg adjustment was used. BiomaRt getBM (version 2.56.1) was used to retrieve entrezgene ids and hgnc symbols for each gene from the BioMart database.

The expression patterns of the top 5,000 most significant genes identified by the LRT were clustered using DEGreport (version 1.36.0) to identify sets of genes with similar expression patterns across the samples. GO and enrichment analysis were performed using clusterProfiler enrichGO (version 4.8.2) or tmod (version 0.50.13), as specified, using all significant DEGs (adjusted *p* < 0.05) against a background of all genes after filtering.

DISCO analysis was performed using the disco package (version 0.6). The input to the DISCO function was all matched orthologous genes found in the significant DEG sets for untreated vs. GFP high and untreated vs. GFP^−^ identified in the DEseq2 Wald test. The BiomaRt getLDS (version 2.56.1) function was used to pair orthologous mouse and human genes. The resulting DISCO score is based on the adjusted *p* value, the magnitude of the log_2_FC, and the direction of the expression change, as described in Domaszewska et al.^21^

To calculate total reads mapped to saRNA genomic and subgenomic transcripts, trimmed reads were aligned to the first 7,524 nt of VEEV saRNA (for genomic-specific transcripts) or the region between 7,586 and 8,415 comprising an EGFP open reading frame and 3′ UTR (for subgenomic transcripts) using bowtie2 and samtools. Relative expression ratios between subgenomic and genomic RNAs were calculated using normalizing total mapped reads to transcript length.

All graphs were made using ggplot2 (version 3.4.2) apart from heatmaps, which were generated using aheatmap from NMF (version 0.26).

### General statistical analysis

Data were collected and analyzed using R (version 4.3.1), Microsoft Excel (2016) and GraphPad Prism (version 7.04). All assays were performed with a minimum n number of three. Graphs depict the mean with SE unless stated otherwise.

## Data and code availability

The dataset has been uploaded to ArrayExpress.

## Acknowledgments

The authors thank Lesley Rawlinson for lab management. For the purpose of open access, the author has applied a Creative Commons Attribution (CC BY) license to any Author Accepted Manuscript version arising. This work was supported by a 10.13039/100010269Wellcome Trust (UK) (109056/Z/15/A) studentship to R.D.B. Support for the project came from the Vaccine Hub. J.S.T. and R.J.S. are supported by an NIHR BRC award to 10.13039/100013216Imperial College (UK).

## Author contributions

R.D.B., visualization, investigation, writing – original draft, writing – review & editing. J.S.T., writing – original draft, writing – review & editing. R.P., investigation. P.F.M., supervision, conceptualization. R.J.S., conceptualization, supervision, writing – review & editing.

## Declaration of interests

The authors declare no competing interests.

## Supplemental information

Document S1. Figures S1–S3

Document S2. Article plus supplemental information

## References

[1] Pepini T., Pulichino A.M., Carsillo T., Carlson A.L., Sari-Sarraf F., Ramsauer K., Debasitis J.C., Maruggi G., Otten G.R., Geall A.J. (2017). Induction of an IFN-Mediated Antiviral Response by a Self-Amplifying RNA Vaccine: Implications for Vaccine Design. J. Immunol..

[2] Guy C., Bowie A.G. (2022). Recent insights into innate immune nucleic acid sensing during viral infection. Curr. Opin. Immunol..

[3] Brito L.A., Chan M., Shaw C.A., Hekele A., Carsillo T., Schaefer M., Archer J., Seubert A., Otten G.R., Beard C.W. (2014). A Cationic Nanoemulsion for the Delivery of Next-generation RNA Vaccines. Mol. Ther..

[4] Shirota H., Petrenko L., Hong C., Klinman D.M. (2007). Potential of transfected muscle cells to contribute to DNA vaccine immunogenicity. J. Immunol..

[5] Hassett K.J., Rajlic I.L., Bahl K., White R., Cowens K., Jacquinet E., Burke K.E. (2024). mRNA vaccine trafficking and resulting protein expression after intramuscular administration. Mol. Ther. Nucleic Acids.

[6] Fu T.M., Ulmer J.B., Caulfield M.J., Deck R.R., Friedman A., Wang S., Liu X., Donnelly J.J., Liu M.A. (1997). Priming of cytotoxic T lymphocytes by DNA vaccines: requirement for professional antigen presenting cells and evidence for antigen transfer from myocytes. Mol. Med..

[7] Li C., Lee A., Grigoryan L., Arunachalam P.S., Scott M.K.D., Trisal M., Wimmers F., Sanyal M., Weidenbacher P.A., Feng Y. (2022). Mechanisms of innate and adaptive immunity to the Pfizer-BioNTech BNT162b2 vaccine. Nat. Immunol..

[8] Khodabukus A., Prabhu N., Wang J., Bursac N. (2018). In Vitro Tissue-Engineered Skeletal Muscle Models for Studying Muscle Physiology and Disease. Adv. Healthc. Mater..

[9] Garlepp M.J., Chen W., Tabarias H., Baines M., Brooks A., McCluskey J. (1995). Antigen processing and presentation by a murine myoblast cell line. Clin. Exp. Immunol..

[10] Shahini A., Vydiam K., Choudhury D., Rajabian N., Nguyen T., Lei P., Andreadis S.T. (2018). Efficient and high yield isolation of myoblasts from skeletal muscle. Stem Cell Res..

[11] Thorley M., Duguez S., Mazza E.M.C., Valsoni S., Bigot A., Mamchaoui K., Harmon B., Voit T., Mouly V., Duddy W. (2016). Skeletal muscle characteristics are preserved in hTERT/cdk4 human myogenic cell lines. Skelet. Muscle.

[12] Mamchaoui K., Trollet C., Bigot A., Negroni E., Chaouch S., Wolff A., Kandalla P.K., Marie S., Di Santo J., St Guily J.L. (2011). Immortalized pathological human myoblasts: towards a universal tool for the study of neuromuscular disorders. Skelet. Muscle.

[13] Stadler G., Chen J.C., Wagner K., Robin J.D., Shay J.W., Emerson C.P., Wright W.E. (2011). Establishment of clonal myogenic cell lines from severely affected dystrophic muscles - CDK4 maintains the myogenic population. Skelet. Muscle.

[14] Zhu C.H., Mouly V., Cooper R.N., Mamchaoui K., Bigot A., Shay J.W., Di Santo J.P., Butler-Browne G.S., Wright W.E. (2007). Cellular senescence in human myoblasts is overcome by human telomerase reverse transcriptase and cyclin-dependent kinase 4: consequences in aging muscle and therapeutic strategies for muscular dystrophies. Aging Cell.

[15] Lucey B.P., Nelson-Rees W.A., Hutchins G.M. (2009). Henrietta Lacks, HeLa cells, and cell culture contamination. Arch. Pathol. Lab Med..

[16] Liu Z., Chen O., Wall J.B.J., Zheng M., Zhou Y., Wang L., Vaseghi H.R., Qian L., Liu J. (2017). Systematic comparison of 2A peptides for cloning multi-genes in a polycistronic vector. Sci. Rep..

[17] Schurch N.J., Schofield P., Gierliński M., Cole C., Sherstnev A., Singh V., Wrobel N., Gharbi K., Simpson G.G., Owen-Hughes T. (2016). How many biological replicates are needed in an RNA-seq experiment and which differential expression tool should you use?. RNA.

[18] Conesa A., Madrigal P., Tarazona S., Gomez-Cabrero D., Cervera A., McPherson A., Szcześniak M.W., Gaffney D.J., Elo L.L., Zhang X., Mortazavi A. (2016). A survey of best practices for RNA-seq data analysis. Genome Biol..

[19] McDougal M.B., De Maria A.M., Ohlson M.B., Kumar A., Xing C., Schoggins J.W. (2023). Interferon inhibits a model RNA virus via a limited set of inducible effector genes. EMBO Rep..

[20] Reynaud J.M., Kim D.Y., Atasheva S., Rasalouskaya A., White J.P., Diamond M.S., Weaver S.C., Frolova E.I., Frolov I. (2015). IFIT1 Differentially Interferes with Translation and Replication of Alphavirus Genomes and Promotes Induction of Type I Interferon. PLoS Pathog..

[21] Domaszewska T., Scheuermann L., Hahnke K., Mollenkopf H., Dorhoi A., Kaufmann S.H.E., Weiner J. (2017). Concordant and discordant gene expression patterns in mouse strains identify best-fit animal model for human tuberculosis. Sci. Rep..

[22] Russell A.B., Trapnell C., Bloom J.D. (2018). Extreme heterogeneity of influenza virus infection in single cells. Elife.

[23] Gilleron J., Querbes W., Zeigerer A., Borodovsky A., Marsico G., Schubert U., Manygoats K., Seifert S., Andree C., Stöter M. (2013). Image-based analysis of lipid nanoparticle–mediated siRNA delivery, intracellular trafficking and endosomal escape. Nat. Biotechnol..

[24] Lundstrom K. (2005). Biology and application of alphaviruses in gene therapy. Gene Ther..

[25] Ganini D., Leinisch F., Kumar A., Jiang J., Tokar E.J., Malone C.C., Petrovich R.M., Mason R.P. (2017). Fluorescent proteins such as eGFP lead to catalytic oxidative stress in cells. Redox Biol..

[26] Roby J.A., Clarke B.D., Khromykh A.A. (2014). Loop de loop: viral RNA evades IFIT1 targeting. Trends Microbiol..

[27] Hyde J.L., Gardner C.L., Kimura T., White J.P., Liu G., Trobaugh D.W., Huang C., Tonelli M., Paessler S., Takeda K. (2014). A Viral RNA Structural Element Alters Host Recognition of Nonself RNA. Science.

[28] Goubau D., Deddouche S., Reis e Sousa C. (2013). Cytosolic sensing of viruses. Immunity.

[29] Karikó K., Buckstein M., Ni H., Weissman D. (2005). Suppression of RNA recognition by Toll-like receptors: the impact of nucleoside modification and the evolutionary origin of RNA. Immunity.

[30] Wang Z., Jacobus E.J., Stirling D.C., Krumm S., Flight K.E., Cunliffe R.F., Mottl J., Singh C., Mosscrop L.G., Santiago L.A. (2023). Reducing cell intrinsic immunity to mRNA vaccine alters adaptive immune responses in mice. Mol. Ther. Nucleic Acids.

[31] Kimura T., Nakayama K., Penninger J., Kitagawa M., Harada H., Matsuyama T., Tanaka N., Kamijo R., Vilcek J., Mak T.W. (1994). Involvement of the IRF-1 transcription factor in antiviral responses to interferons. Science.

[32] Brien J.D., Daffis S., Lazear H.M., Cho H., Suthar M.S., Gale M., Diamond M.S. (2011). Interferon regulatory factor-1 (IRF-1) shapes both innate and CD8(+) T cell immune responses against West Nile virus infection. PLoS Pathog..

[33] Maloney N.S., Thackray L.B., Goel G., Hwang S., Duan E., Vachharajani P., Xavier R., Virgin H.W. (2012). Essential cell-autonomous role for interferon (IFN) regulatory factor 1 in IFN-γ-mediated inhibition of norovirus replication in macrophages. J. Virol..

[34] Nair S., Poddar S., Shimak R.M., Diamond M.S. (2017). Interferon Regulatory Factor 1 Protects against Chikungunya Virus-Induced Immunopathology by Restricting Infection in Muscle Cells. J. Virol..

[35] Blakney A.K., McKay P.F., Bouton C.R., Hu K., Samnuan K., Shattock R.J. (2021). Innate Inhibiting Proteins Enhance Expression and Immunogenicity of Self-Amplifying RNA. Mol. Ther..

[36] Shaw A.E., Hughes J., Gu Q., Behdenna A., Singer J.B., Dennis T., Orton R.J., Varela M., Gifford R.J., Wilson S.J., Palmarini M. (2017). Fundamental properties of the mammalian innate immune system revealed by multispecies comparison of type I interferon responses. PLoS Biol..

[37] Pollock K.M., Cheeseman H.M., Szubert A.J., Libri V., Boffito M., Owen D., Bern H., O'Hara J., McFarlane L.R., Lemm N.M. (2022). Safety and immunogenicity of a self-amplifying RNA vaccine against COVID-19: COVAC1, a phase I, dose-ranging trial. EClinicalMedicine.

[38] Blakney A.K., Zhu Y., McKay P.F., Bouton C.R., Yeow J., Tang J., Hu K., Samnuan K., Grigsby C.L., Shattock R.J., Stevens M.M. (2020). Big Is Beautiful: Enhanced saRNA Delivery and Immunogenicity by a Higher Molecular Weight, Bioreducible, Cationic Polymer. ACS Nano.

