# Supplementary material for: A sort and sequence approach to dissect heterogeneity of response to a self-amplifying RNA vector in a novel human muscle cell line
Source: Mol Ther Nucleic Acids. 2024 Nov 26;36(1):102400. doi: 10.1016/j.omtn.2024.102400 (PMC11700297; doi:10.1016/j.omtn.2024.102400)
Supplement: Document S1. Figures S1–S3 [file mmc1.pdf]

OMTN, Volume *36*

Supplemental information

A sort and sequence approach to dissect heterogeneity of response to a self-amplifying RNA vector in a novel human muscle cell line

Rachel D. Barton, John S. Tregoning, Ziyin Wang, Daniel Gonçalves-Carneiro, Radhika Patel, Paul F. McKay, and Robin J. Shattock

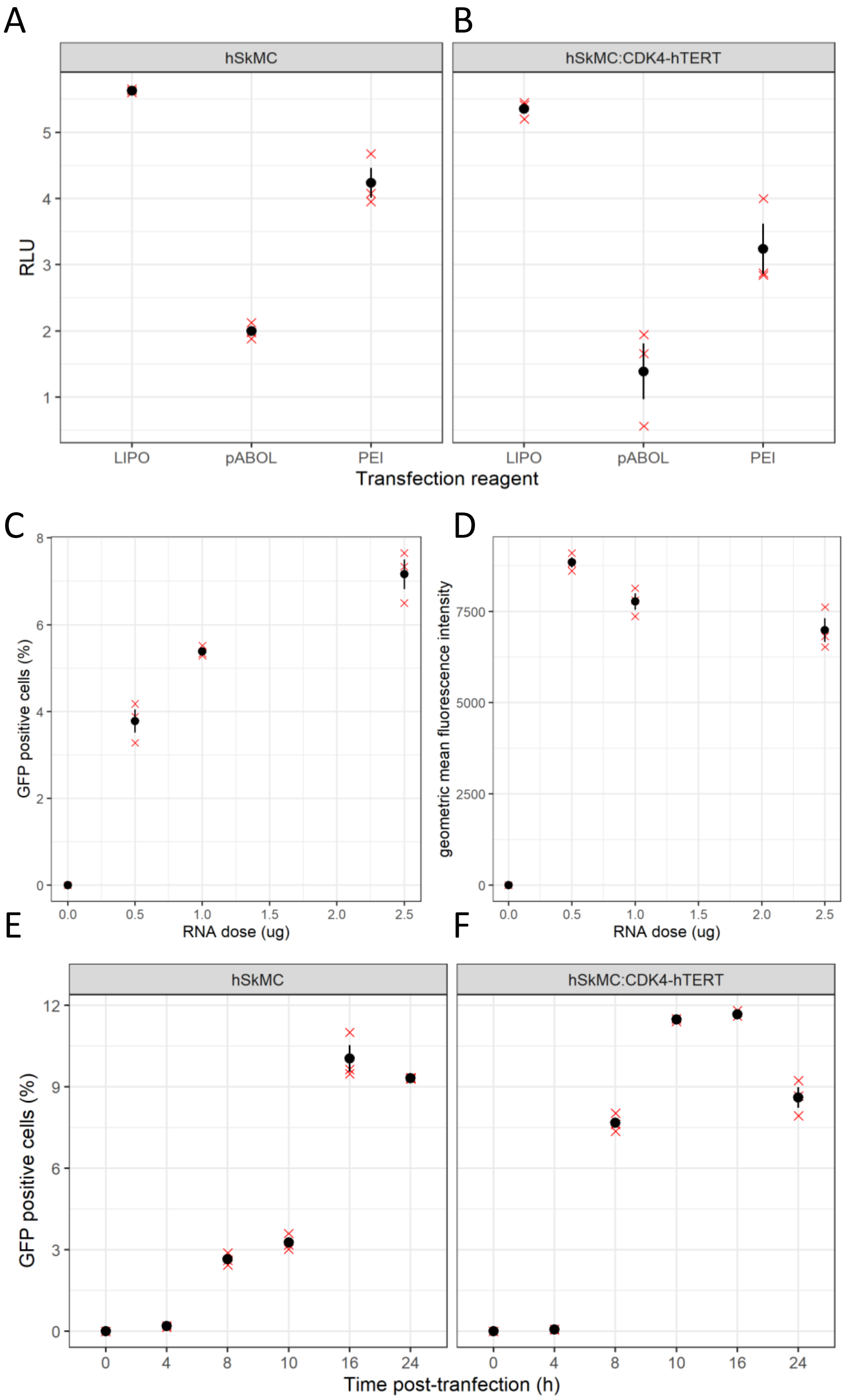

Figure S1. Optimisation of dose and polymer. To test different transfection reagents primary (A) and immortalised (B) human myoblasts were transfected with equal quantities of VEEV Fluc saRNA. 24 hours later, luciferase assay performed to assess expression. To optimise VEEV saRNA dose in human immortalised muscle cells we used a VEEV expressing GFP and flow cytometry. (C) Percentage of GFP-positive cells per sample. (D) Geometric mean fluorescence intensity. Percentage of GFP positive primary (E) and immortalised (F) human muscle cells at various intervals post transfection with 1ng/µL VEEV GFP to determine the optimum incubation time for FACS. Red crosses represent individual replicates, black dots represent the mean, and the error bars show the standard error.

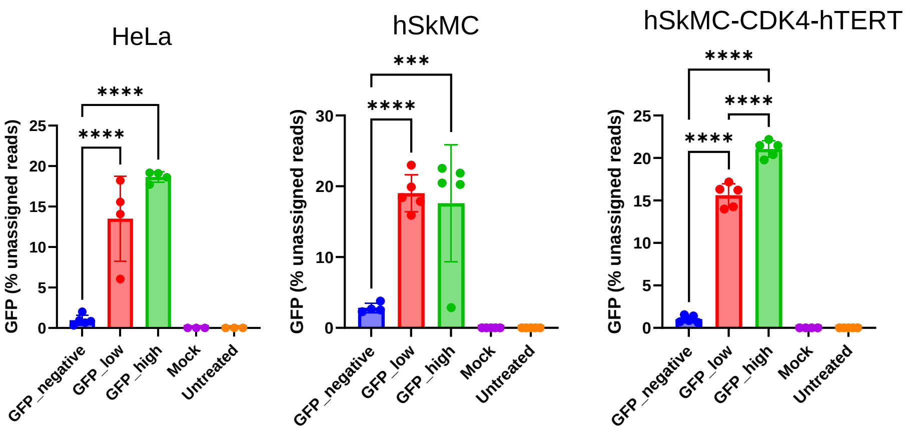

Figure S2. GFP as proportion of unmapped reads. GFP reads were mapped as a percentage of total unmapped reads in sorted HeLa (A), hSkMC (B) and hSkMC-CDK4-hTERT (C) cells.

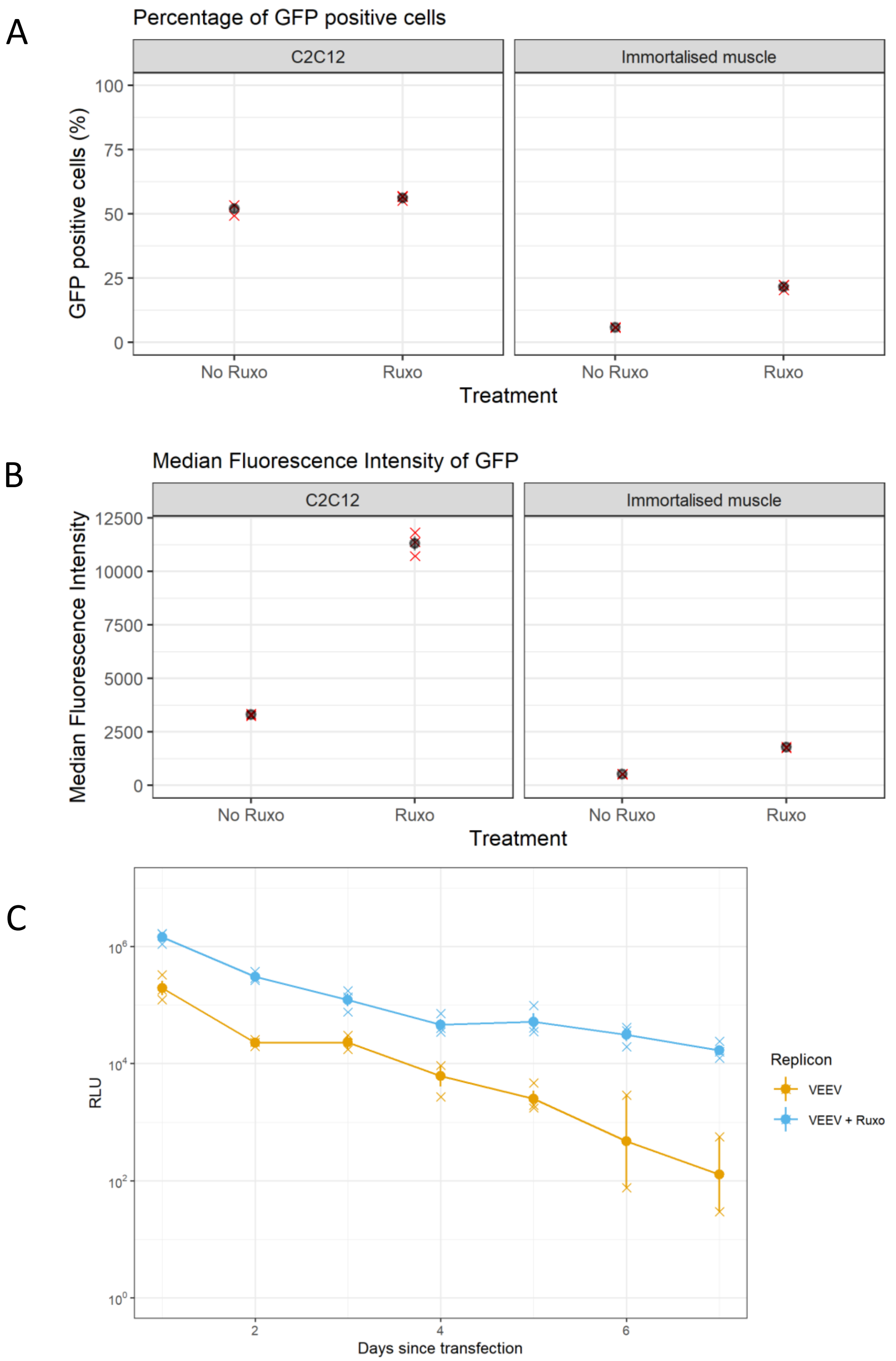

Figure S3. Ruxolitinib treatment increases luciferase expression. C2C12 cells and immortalised human muscle cells were transfected with Fluc GFP with and without 30 ng/µL Ruxolitinib (Ruxo) and the percentage of GFP positive cells (A) and median fluorescence intensity (B) were measured after 24 hours. Human muscle cells were transfected with Fluc VEEV with and without 30 ng/µL Ruxolitinib (Ruxo). Luciferase expression was measured 24 hours after transfection (C).
